# Annotations

**Published:** 1899-09-16

**Authors:** 


### Annotations.

### Patented Germs.

The idea of a germ is popularly so closely associated with disease that it is seldom realised that some varieties serve useful ends, and are used for commercial purposes and to contribute to man's comfort and enjoyment. Such, however, is the case, and each year the traffic in germs assumes greater proportions. The nitrifying organisms of the soil, and many special varieties which attach themselves to the rootlets of certain plants, and which are essential to the perfect growth of these plants, have been successfully isolated, and are now retailed to agriculturists as fertilisers. Certain yeasts also, and other fermentative germs are grown in bulk, and have a high commercial value. In America, however, a step further has been taken, and patents have been granted conferring on certain persons the exclusive right to utilise the services of particular germs. It has long been known that the flavour and aroma of butter and cheese are due to ethers and esters elaborated by micro-organisms, and that the failure to secure a satisfactory article often depends upon the defective growth of these varieties or to the presence of an unfavourable species. Since it has been possible to isolate and grow the required species the manufacturer has been enabled to secure butter and cheese of standard flavour, all extraneous organisms being eliminated by the use of sterilised milk. It is for this process that patents have been granted, and consumers of dairy produce will now be enabled to see that they secure the right brand. The attempt which was recently made to obtain protection for diphtheria antitoxin would point to the fact that bacterial products may some day come under the care of the Patent Office. We doubt, however, whether there is any probability of patents being granted for pathogenic bacteria, though it is possible that as man's knowledge of and control over these varieties extends these, too, may be found to subserve some useful purpose.

### The Waste of Town's Water.

It is stated that in the area supplied by the East London Water Company over 50,000,000 gallons of water are allowed to run to waste every day, or more than would be required to give two days' supply at 20 gallons per head to the whole of the population served by the company. This waste is principally the result of bad fittings, of allowing garden hose to run all night, and of culpable negligence in letting domestic taps do the same. What is clear is that unless a stop is put to such extravagance, the new measures recently taken for providing an increased supply, and for arranging means of intercommunication between the companies, will but become a still more efficient means for educating the people in wastefulness. The question then is, Who is to put the law in force and to see that the water is not allowed to run to waste? Clearly this is the duty of the company, and thus the company is really responsible for the waste about which it makes such complaints. We have hopes, however, that the introduction of pipes of intercommunication between the different companies will lead to better arrangements being made. So long as in face of drought all that they had to do was to diminish the hours of supply it was the consumer rather than the company that suffered when the supply ran short. If, however, when the water fails, the company has to purchase a supply from other sources, it will be worth while to take trouble to prevent it failing, which we have no doubt will be done. The prevention of waste is chiefly a matter of constant inspection and enforcement of the law as to fittings. All this means money; but it can be done, and no doubt it will be done when, as the penalty of neglect, the company has to buy water from elsewhere.

### Microbes in the Air of Schools.

In a paper read at Portsmouth Dr. Atkinson Dove detailed the results of a series of investigations which he had made as to the bacteriology of the air of school-rooms. These investigations had been undertaken in elementary schools by the permission of the medical officer to the School Board for London, and the examination of the specimens obtained was made in the bacteriological laboratory of King's College. A large number of micro-organisms were subcultured and recognised, but no pathogenic microbes were found. The most striking thing demonstrated was the enormous increase in the number of microbes present whenever the children moved about or in any way disturbed the air, and by inference the dust; and it is in regard to this question of dust that Dr. Dove's investigation seems to teach a lesson. "That schools should be systematically cleaned and swept out every night after the children leave does not," he says, "seem to be sufficiently insisted upon. I say advisedly systematically; the use of the brush, while giving a cleanly look to the room, throws up an innumerable number of organisms which again settle on the floor, behind the brush, in the crevices, ledges, and walls of the room. . . . . The running of a damp cloth over desks, walls, and floor does infinitely more to sterilise the air than anything we can anticipate from repeated brushings. This is generally recognised in all bacteriological laboratories, hospitals, &c., where pathogenic organisms are expected to exist, and this should be the universal means of removing dust from all interiors." Dusting is in the highest degree unscientific; the damp cloth is the thing. And how simple it all seems! The health of nations lies in the hand of the charwoman.

### The Peculiar People.

Common sense, even in small doses, has a curiously disruptive effect upon creeds founded upon prejudice. Upon the Peculiar People the effect has been disastrous. One of their elders, without having wit enough to sever himself from his co-religionists, seems somehow or another to have become infected by a slight modicum of common sense, just enough to teach him that, however right it may be for grown people to refrain from sending for a doctor when they are themselves ill if they like so to do, it can hardly be right to enforce the same plan on children of tender years. He has been moved by the deformities and disfigurements he sees around him to doubt the efficacy of prayer in mending broken bones, and indeed the number of deaths among the young ones of the People from "natural causes" seems to have made him question whether the tenets of the sect are fully applicable to infants. So when his children had the measles he called in a doctor. This would to most people appear a small thing, and reasonable withal. But it seems not unlikely that it will split the sect. A most satisfactory conclusion.

